# Kidney injury molecule-1 inhibits metastasis of renal cell carcinoma

**DOI:** 10.1038/s41598-021-90919-8

**Published:** 2021-06-04

**Authors:** Jasper C. Lee, Demitra M. Yotis, Ji Yun Lee, Marie A. Sarabusky, Bradly Shrum, Audrey Champagne, Ola Z. Ismail, Elena Tutunea-Fatan, Hon S. Leong, Lakshman Gunaratnam

**Affiliations:** 1 Department of Microbiology and Immunology, Schulich School of Medicine and Dentistry, Western University, London, ON Canada; 2 Matthew Mailing Centre for Translational Transplant Studies, Lawson Health Research Institute, London, ON Canada; 3 Centre de recherche du CHU de Québec-Université Laval, CHU de Québec-Université Laval, Quebec City, QC Canada; 4 Sunnybrook Health Sciences Centre, Toronto, ON Canada; 5 Division of Nephrology, Department of Medicine, Schulich School of Medicine and Dentistry, Western University, Room A10-208, 339 Windermere Road, London, ON N6A 5A5 Canada

**Keywords:** Cancer genomics, Metastasis, Renal cell carcinoma

## Abstract

Metastasis is present in approximately 30% of patients diagnosed with renal cell carcinoma (RCC) and is associated with a 5-year survival rate of < 15%. Kidney injury molecule 1 (KIM-1), encoded by the *HAVCR1* gene, is a proximal tubule cell-surface glycoprotein and a biomarker for early detection of RCC, but its pathophysiological significance in RCC remains unclear. We generated human and murine RCC cell lines either expressing or lacking KIM-1, respectively, and compared their growth and metastatic properties using validated methods. Surprisingly, KIM-1 expression had no effect on cell proliferation or subcutaneous tumour growth in immune deficient (Rag1^−/−^) Balb/c mice, but inhibited cell invasion and formation of lung metastasis in the same model. Further, we show that the inhibitory effect of KIM-1 on metastases was observed in both immune deficient and immune competent mice. Transcriptomic profiling identified the mRNA for the pro-metastatic GTPase, Rab27b, to be downregulated significantly in KIM-1 expressing human and murine RCC cells. Finally, analysis of The Cancer Genome Atlas (TCGA) data revealed that elevated *HAVCR1* mRNA expression in the two most common types of RCC, clear cell and papillary RCC, tumours correlated with significantly improved overall patient survival. Our findings reveal a novel role for KIM-1 in inhibiting metastasis of RCC and suggests that tumour-associated KIM-1 expression may be a favourable prognostic factor.

## Introduction

Metastases are the primary cause of cancer-related morbidity and mortality^1^. Identification of proteins that regulate the metastatic process can be of great clinical significance as predictive biomarkers or therapeutic targets^2^. Renal cell carcinoma (RCC) is the most common form of kidney cancer and accounts for over 90% of all kidney cancers^3^. The prognosis for patients with metastatic RCC is very poor with 5-year survival rates of 10–12% in patients with stage IV RCC^4^. Due to a lack of effective screening tests for the early stages of the disease, over 30% of patients present with metastases at the time of diagnosis^5^.

Kidney injury molecule-1 (KIM-1) is a type-1 cell surface glycoprotein expressed in over 90% of human RCC tissue samples and is encoded by the *HAVCR1* gene^6,7^. KIM-1 normally functions as a phosphatidylserine receptor that transforms proximal tubular epithelial cells (PTECs) into semi-professional phagocytic cells that can recognize and engulf apoptotic and necrotic cells, and is a highly upregulated marker of renal injury in the proximal tubule^8–11^.

KIM-1 is overexpressed in the two most prevalent forms of RCC, clear cell RCC and papillary RCC, and is touted to become a novel non-invasive biomarker for the early diagnosis of the disease through the detection of the shed ectodomain in the urine and blood of patients^7,12^. In contrast, KIM-1 is absent in normal kidney tissue, and in patients with RCC tumours testing positive for KIM-1 expression before nephrectomy, showed either complete or marked reduction in KIM-1 expression post-nephrectomy^6,12^. The biological significance of KIM-1 overexpression in RCC remains unclear. While some groups have proposed that KIM-1 may drive RCC cell proliferation and tumour progression, our data is not congruent^13–15^. KIM-1 expression by RCC cells is associated with increased levels of TGF-β, which has been shown to promote metastatic invasion in independent studies^16,17^. We previously demonstrated that KIM-1 expression on injured renal proximal tubular cells inhibits Gα_12_ activation downstream signalling via RhoA—a small GTPase implicated in invasion and metastasis^18^. The precise role KIM-1 plays in RCC pathogenesis remains poorly understood. Here, we sought to determine the pathophysiologic and prognostic significance of tumour-associated KIM-1 in RCC using validated human and murine models, as well as The Cancer Genome Atlas (TCGA) dataset, respectively.

## Materials and methods

### Mice

Female *wild-type* (WT) BALB/c mice (Charles River Laboratory, Wilmington, MA) and immune-deficient C129-Rag (Rag1^−/−^) mice (Jackson Laboratory, Bar Harbor, ME) were used in the experimental metastasis model. All animal protocols and experiments were approved by Western University’s Animal Use Subcommittee in compliance with the guidelines set by the Canadian Council of Animal Care. This study was carried out in compliance with the ARRIVE guidelines.

### RCC cell lines and cell culture

Human 786-O renal cell adenocarcinoma (CRL-1932) and murine renal adenocarcinoma (Renca) (CRL-2947) cells were purchased from American Type Tissue Collection (ATCC). We and others have previously been reported that 786-O cells endogenously express functional KIM-1^19,20^. Renca cells were cultured in RPMI-1640 medium, supplemented with 25 mM HEPES (Lonza, Walkersville, MD), 10% fetal bovine serum (FBS; Invitrogen, Carlsbad, CA), 2 mM l-glutamine (ThermoFisher Scientific, Waltham, MA), 1 mM sodium pyruvate (ThermoFisher Scientific, Waltham, MA), 0.1 mM non-essential amino acids (ThermoFisher Scientific, Waltham, MA), and 5% penicillin streptomycin (PS; Invitrogen). Stable Renca cell lines were maintained with 2 mM puromycin dihydrochloride (Sigma-Aldrich, St. Louis, MO). 786-O cells were cultured in complete DMEM medium (Wisent Bioproducts, Saint-Jean Baptiste, QC), supplemented with, 10% FBS, and 5% PS. Stable 786-O cell lines were maintained with 400 μγ/ml geneticin sulfate (G418; Santa Cruz Biotechnology, Santa Cruz, CA) in a humidified incubator (5% CO_2_, 37 °C).

### Stable expression of KIM-1

Lentivirus open reading frame (ORF) particles containing a vector with the murine KIM-1 gene, *HAVCR1* and a puromycin resistance gene (MR203831L3V; Origene, Rockville, MD) were used to transduce Renca cells (herein referred to as KIM-1^pos^ Renca). A lentivirus ORF particle containing the same vector but lacking the KIM-1 transcript (PS100092V; Origene) was used as a negative control (KIM-1^neg^ Renca). Polyclonal cells were selected post transfection, and stable cell lines were isolated via complete DMEM medium supplemented with puromycin (according to manufacturer’s recommendations).

### shRNA knockdown of endogenous KIM-1

Lentiviral particles containing three human KIM-1-specific constructs encoding shRNA (sc-61691-V; Santa Cruz Biotechnology) were used to knockdown KIM-1 in the 786-O cell line (herein referred to as 786-O-shKIM-1). Control transduction was done using scrambled shRNA lentiviral particles (786-O-shControl) (sc-108080; Santa Cruz Biotechnology). Polyclonal clones were selected post transfection, and stable cell lines expressing the respective shRNA constructs were maintained in puromycin dihydrochloride (Santa Cruz Biotechnology)^21^.

### Invasion assay

Transwell culture plates with 8.0-μm-pore-size polycarbonate membrane filter inserts with 6.5 mm diameter (Corning, NY) were used to perform invasion assays. Plate wells were filled with serum-free media (SFM) or complete media with 10% FBS (Renca in RPMI; 786-O in DMEM). Renca cells were resuspended in SFM at a concentration of 2 × 10^5^ cells/ml. 786-O cells were resuspended in SFM at a concentration of 1 × 10^5^ cells/mL. Cells were seeded in the Transwell inserts coated with 200 µL of Matrigel (1:100 dilution in SFM; BD Biosciences, NJ) 12 h prior to seeding of cells. The assembled plates were incubated for 24 h in a 37 °C, 5% (v/v) CO_2_ incubator. After incubation, insert membranes were dyed with eosin (Sigma-Aldrich, Oakville CA) and toluidine blue (Sigma-Aldrich, Oakville CA). Light microscopy was used to quantify the migrated and invaded cells (Leica Microsytems).

### Experimental metastasis model

Renca cells were injected (5 × 10^5^ cells/mouse) into the lateral tail veins of 8–10-week-old female WT BALB/c mice. To study whether KIM-1-mediated inhibition of metastasis is dependent on adaptive immunity, Renca or 786-O cells were instead injected into the tail veins of immune-deficient BALB/c (Rag1^−/−^) mice in the same manner as described above. All mice were sacrificed at day 17 post injection.

### Western blot

Cell lysates from 786-O or Renca cell lines were collected with 4% (w/v) sodium dodecyl sulfate (SDS; Bio Basic Inc.) in 1 × phosphate buffered saline. Total protein was quantified using the Pierce BCA Protein Assay Kit (Thermo Fisher Scientific, Rockford, IL). The lysates were boiled for 5 min at 95 °C to denature proteins. Samples were separated by SDS-PAGE, and transferred to polyvinylidene difluoride membranes (Millipore, Billerica, MA) for 50 min at 90 V. Membranes were blocked with 3% (w/v) BSA (bovine serum albumin; Bio Basic Cat No. AD0023) in TBST (Tris-buffered saline, 0.2% Tween-20) for 30 min and then incubated overnight at 4 °C with anti-murine KIM-1 goat primary antibody (dilution 1:2000; Cat No. AF1817, R&D Systems, Minneapolis, MN) or anti-human monoclonal KIM-1 antibody (AKG, a kind gift from Dr. Bonventre) and anti-GAPDH monoclonal monoclonal or I-19 anti-β-Actin polyclonal antibody (dilution 1:1; Cat No. Sc-32233, and Cat No. Sc-1616, Santa Cruz Biotechnology). After incubating with the appropriate horse radish peroxidase (HRP)-conjugated secondary antibodies, the proteins were visualized using chemiluminescent HRP substrate (Millipore, Billerica, MA). The images for Renca cell lines were captured and analyzed using the Licor C-digit imaging device and Image Studio Lite, respectively. Western blots of human RCC cell lines (769-P and 786-O shControl and shKIM-1) reactive bands were observed by Super Signal West Pico (ThermoFisher Scientific, Waltham, MA). Blots were exposed onto blue X-Ray film and developed using the Kodak M35A-X-OMAT. Resulting protein bands were scanned using a Brother Scanner from the film (Brother Electronics), generating a grey scale image.

### Visualization of metastatic nodules

Lungs were dyed via intra-tracheal injection of 15% India black ink (Superball, Statesville NC). Lungs were excised and placed in a beaker of distilled water for 5 min to wash off excess ink. Lungs were then transferred into Fekete’s solution and incubated overnight at 4 °C before being washed with 1 × PBS. Metastases were counted under a low power light microscope (Leica Microsystems, etc.).

### RNA sequencing

RNA extraction and cDNA library synthesis were performed following standard protocols. Libraries were sequenced using the Illumina NextSeq 500 sequencer (Illumina Inc., San Diego, CA). RNA-sequencing (RNAseq) data were analyzed using the Partek Flow software (Partek Inc., St. Louis, MO) to compare differences in gene expression between KIM-1^pos^ and KIM-1^neg^ Renca cells. Gene pathway and gene set enrichment were performed to determine biological pathways and specific enriched genes relevant to invasion and metastasis. Significant enriched genes of KIM-1^pos^ vs. KIM-1^neg^ cell lines were arranged into a heat map. Enriched genes were filtered through TMM normalization with reads less than 100 excluded. Further stringencies for significance and fold change were applied to only account for genes with p-values ≤ 0.05, and fold changes of − 1.5 to 1.5. All analyses for heat maps and enriched gene lists were performed using Partek Flow Inc. Technology.

### TCGA RNAseq analyses

*HAVCR1* (KIM-1) mRNA expression in human RCC was observed using The Cancer Genome Atlas (TCGA) RNA-sequencing for Pan-Kidney KIPAN database; including patient information from KIRC (Kidney Clear Cell Carcinoma), KIRP (Kidney Papillary Carcinoma) and KICH (Kidney Chromophobe Carcinoma) TCGA databases combined. KIPAN primary data were extracted from http://gdac.broadinstitute.org/ with all transcripts quantified and normalized by RSEM Software^22^. Raw KIPAN primary data was organized into a working Excel file to analyse all clinical and survival patient data. KIPAN data was filtered based off *HAVCR1* expression being elevated in KIRC and KIRP, but not in KICH. Using the working excel file, KIPAN data was filtered to analyzed KIRC and KIRP combined, as well as KIRC alone and KIRP alone. Differences in *HAVCR1* mRNA expression between matched normal adjacent tissues vs. tumour tissues were analyzed using Normality and Lognormality statistics for KIRC and KIRP combined, KIRC alone and KIRP alone. Furthermore, differences in *HAVCR1* mRNA expression between non-matched normal adjacent tissues vs. tumour tissues were analyzed using Normality and Lognormality statistics for KIRC and KIRP combined, KIRC alone and KIRP alone. Lastly, the ratio calculated between matched normal adjacent tissues vs tumour tissues were analyzed using Normality and Lognormality statistics for KIRC and KIRP combined, KIRC alone and KIRP alone. Tumour stage vs *HAVCR1* mRNA expression was compared using Kruskal–Wallis statistical analysis for KIRC and KIRP combined, KIRC alone and KIRP alone. Survival curves were used to compare differences between low and high (L/H) *HAVCR1* expressors vs overall patient survival. Survival curves for both female and male low and high (L/H) *HAVCR1* expressors vs overall patient survival were analyzed to compare differences between sexes. All survival analyses were based off patients’ most recent follow up (months) and performed using Kaplan–Meier statistics. Low and high (L/H) 50%, and 30% *HAVCR1* expressors were examined for KIRC and KIRP combined, as well for KIRC alone and KIRP alone. Clinical characteristics of top and bottom L/H 50% expressors for *HAVCR1* are listed in Table 1.

**Table 1:** Clinical characteristics of the top and bottom *HAVCR1* (KIM-1) mRNA expressing patients in the KIRC and KIRP TCGA databases.

Variable	Top 50%	Bottom 50%
**Disease code**		
Kidney clear cell carcinoma	273 (61.48)	259 (68.51)
Kidney papillary carcinoma	171 (38.51)	119 (26.8)
Age	60.96832579	60.82493369
**Sex**		
Male	298 (67.11)	117 (30.95)
Female	146 (32.88)	261 (69.04)
**Race**		
White	364 (81.98)	303 (80.15)
American Indian or Alaskan	2 (0.45)	0 (0)
Black or African American	53 (11.93)	64 (16.93)
Asian	10 (2.25)	4 (1.05)
n/a	15 (3.37)	7 (1.85)
**Stage**		
Stage I	252 (56.75)	187 (49.47)
Stage II	43 (9.68)	35 (9.25)
Stage III	93 (20.94)	81 (21.42)
Stage IV	42 (9.45)	57 (15.07)
n/a	14 (3.15)	18 (4.76)
**Pathologic M**		
m0	285 (64.18)	231 (61.11)
m1	39 (8.78)	49 (12.96)
Mx	109 (24.54)	92 (24.33)
n/a	11 (2.47)	6 (1.58)
**Pathologic N**		
n0	154 (34.68)	135 (35.71)
n1	21 (4.72)	19 (5.02)
n2	2 (0.45)	2 (0.52)
Nx	267 (60.13)	221 (58.46)
n/a		1 (0.26)
**Pathologic T**		
t1	29 (6.53)	15 (3.96)
t1a	137 (30.85)	111 (29.36)
t1b	100 (22.52)	74 (19.57)
t2	36 (8.10)	38 (10.05)
t2a	12 (2.70)	7 (1.85)
t2b	4 (0.90)	5 (1.32)
t3	8 (1.8)	6 (1.58)
t3a	77 (17.34)	83 (21.95)
t3b	35 (7.88)	27 (7.14)
t3c	1 (0.22)	2 (0.52)
t4	3 (0.6)	10 (2.64)
Tx	2 (0.45)	
**Histologic grade**		
g1	10 (2.25)	4 (1.05)
g2	123 (27.70)	105 (27.77)
g3	109 (24.54)	97 (25.66)
g4	29 (6.53)	47 (12.43)
gx	1 (0.22)	4 (1.05)
n/a	172 (38.73)	121 (32.01)

### Statistical analysis

Differences in means between KIM-1^pos^ and KIM-1^neg^ groups for all results were analyzed using unpaired two-tailed *t* tests. Statistical significance was defined as *p < 0.05; **p < 0.01; ***p < 0.001. Statistical analyses were performed using the GraphPad Prism, version 8 software (GraphPad Software Inc.; La Jolla, CA).

## Results and discussion

### KIM-1 expression does not alter RCC cell proliferation or tumour growth in vivo

**Figure 1:**
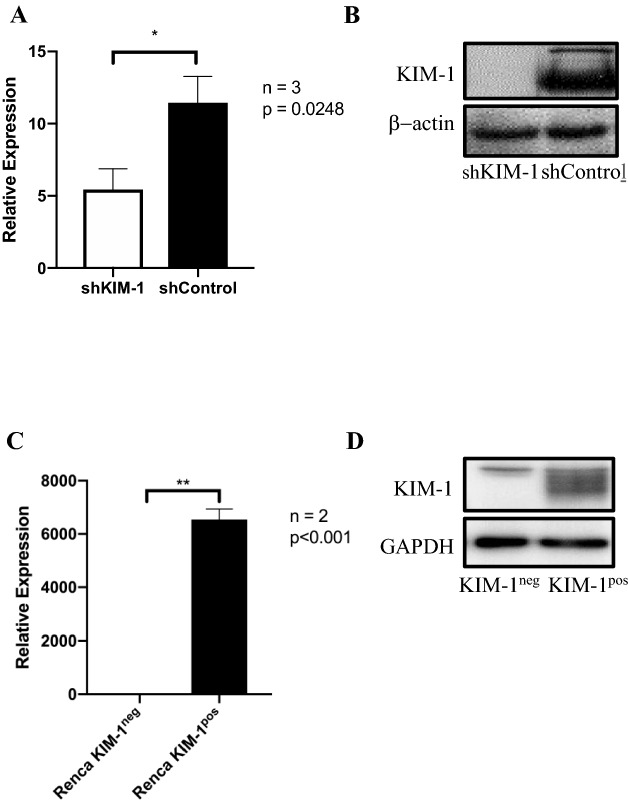
Knockdown of human KIM-1 in 786-O cells and expression if murine KIM-1 in Renca RCC cells. (**A**) 786-O cell lines were transduced using lentivirus encoding either KIM-1 shRNA or a control shRNA plasmid. RT-qPCR of 786-O cell lines normalized to housekeeping gene (GAPDH), showing successful knockdown of KIM-1 mRNA in 786-O-shKIM-1, but not in 786-O-shControl (*p = 0.0248). (**B**) Western Blot analyses confirming KIM-1 protein knockdown in 786-O-shKIM-1, but not 786-O-shControl cell lines. (**C**) Renca cell lines were transduced using lentivirus encoding either KIM-1 expressing (KIM-1^pos^) or non-expressing (KIM-1^neg^) vectors. RT-qPCR of Renca cell lines normalized to housekeeping gene (GAPDH), showing successful upregulation of KIM-1 in Renca KIM-1^pos^, but not Renca KIM-1^neg^ cell lines (**p < 0.001). (**D**) Western blot analyses confirming strong cellular KIM-1 protein expression in Renca KIM-1^pos^, but not in Renca KIM-1^neg^ cell lines. The cropped Western blots images in (**A**) and (**B**) were obtained from the same gel but the resulting membranes were cut and probed with either anti-KIM-1 or anti-GAPDH antibodies for (**A**). The exposures was optimized by the digital imaging system.

**Figure 2:**
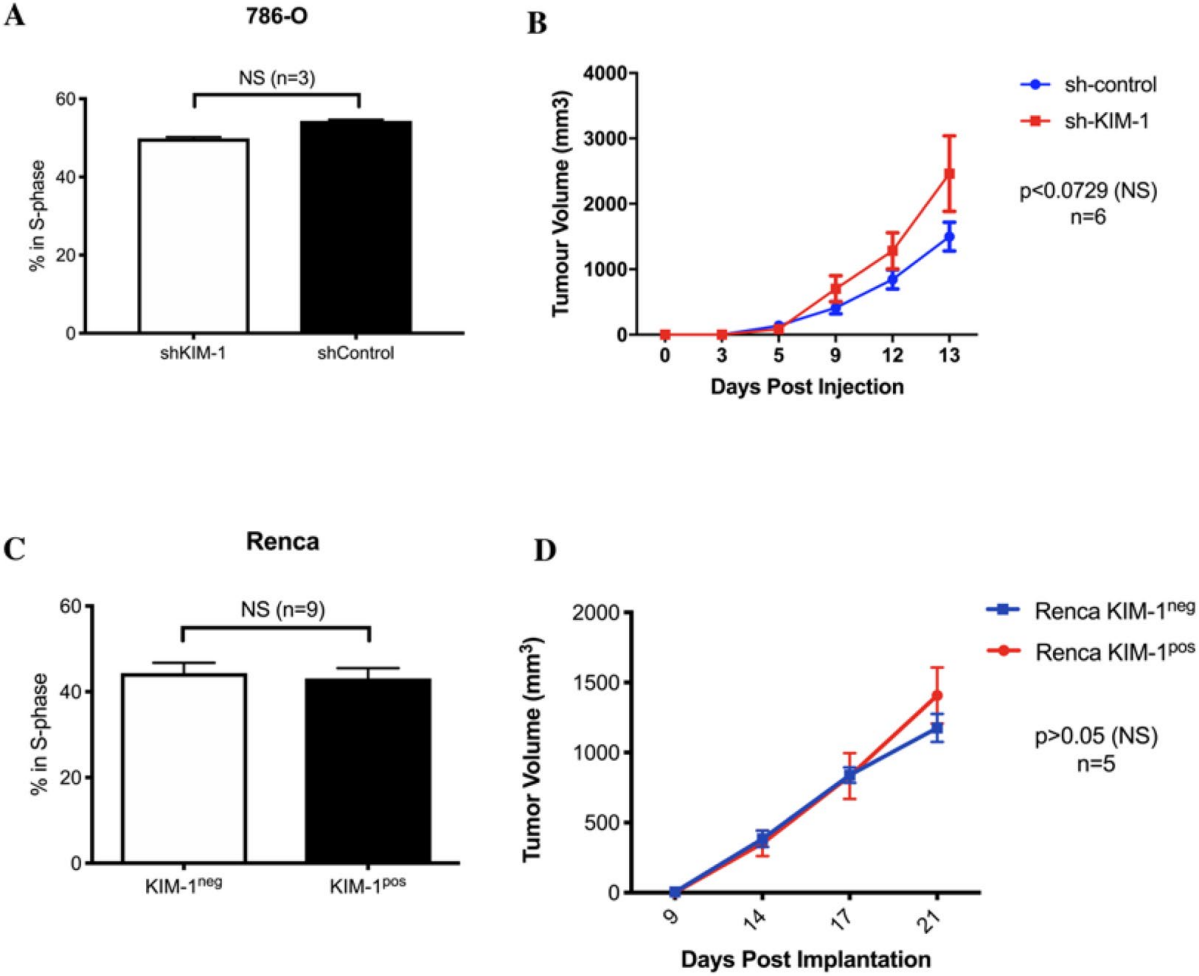
KIM-1 expression does not alter the proliferation or spontaneous generation of tumours by human and murine RCC cells. (**A**) Quantification of proliferation responses of human RCC 786-O-shControl and 786-O-shKIM-1 cell lines using BrdU (Bromodeoxyuridine) assay. Data are represented as mean ± SD of n = 3 independent experiments (p = 0.1000, NS). (**B**) 786-O-shControl and 786-O-shKIM-1 tumour volumes (mm^3^) and pictorial representation after 15 days of incubation in immune deficient Rag1^−/−^ Balb/c mice. Data are represented as mean ± SD of n = 6 independent experiments (p < 0.0729, NS). (**C**) Quantification of proliferation responses of Renca KIM-1^pos^ and Renca KIM-1^neg^ cell lines using BrdU (Bromodeoxyuridine) assay. Data are represented as mean ± SD of n = 9 independent experiments (p = 0.4363, NS). (**D**) Renca KIM-1^pos^ and Renca KIM-1^neg^ tumour volumes (mm^3^) and pictorial representation after 21 days of incubation in immune deficient Rag1^−/−^ Balb/c mice. Data points are represented as mean ± SD of n = 5 independent experiments (p > 0.05, NS).

We explored the role of tumour-associated KIM-1 in RCC using human and murine models. We silenced endogenous KIM-1 in human 786-O cells^23,24^ using shRNA (Fig. 1A,B), and expressed exogenous murine KIM-1 (KIM-1^pos^) in murine Renca (RCC) cells using lentiviruses [Renca cells do not express endogenous KIM-1] (Fig. 1C,D). The effectiveness of knockdown or transfection was assessed at the mRNA and protein level as shown in Fig. 1. Targeted silencing of KIM-1 or KIM-1 overexpression had no effect on RCC cell proliferation in both human (786-O) and murine (Renca) cells in vitro (Fig. 2A,C).

Moreover, we did not observe any significant differences in KIM-1-dependent tumour growth upon subcutaneous implantation of either 786-O or Renca cells into the flanks of immune-deficient (Rag-1^−/−^) Balb/c mice (Fig. 2B,D). Taken together, these data indicate that KIM-1 does not promote RCC tumour growth characteristics.

### KIM-1 expression inhibits RCC cell invasion

**Figure 3:**
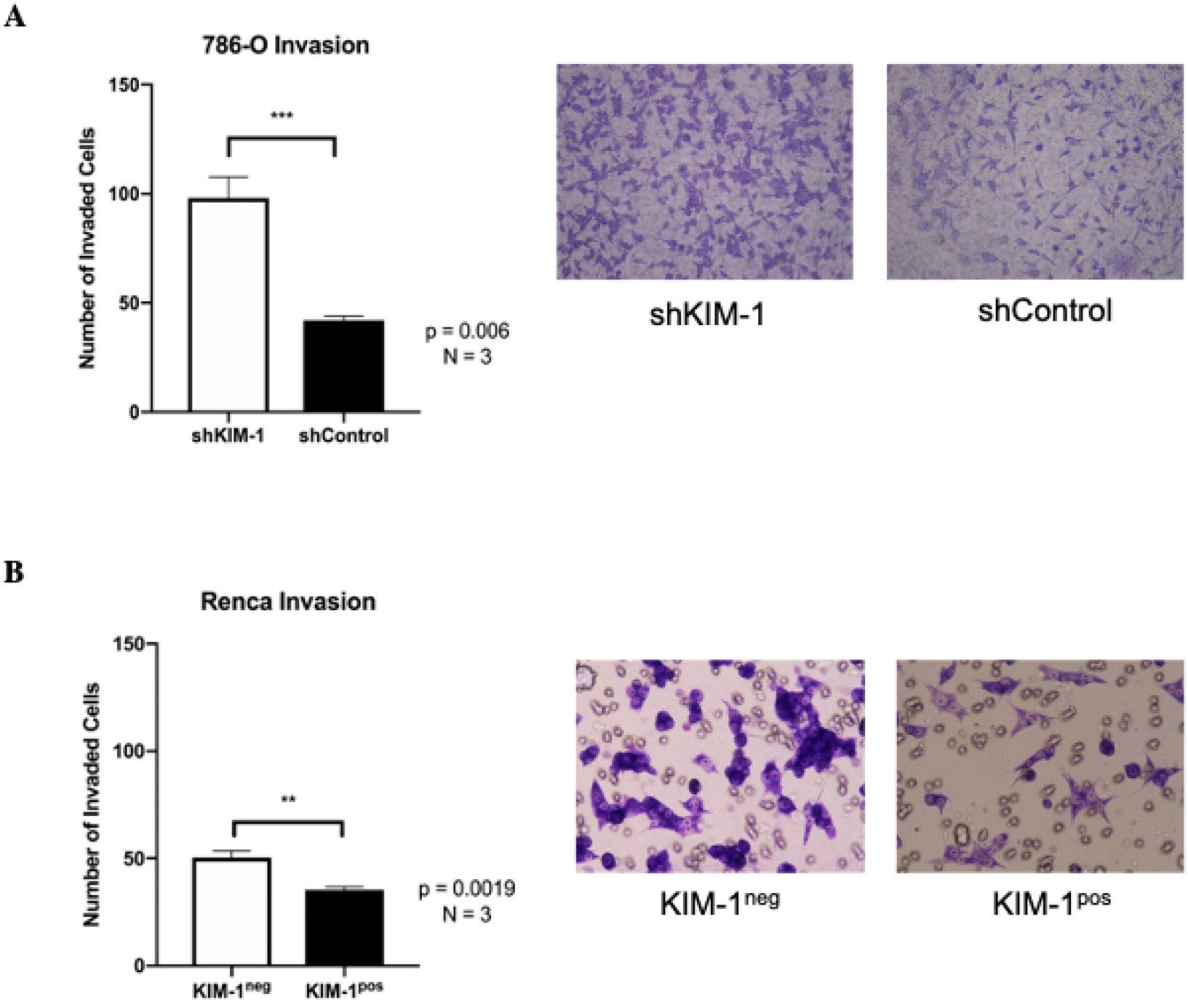
KIM-1 expression inhibits the invasion of human and murine RCC cells in vitro. (**A**) Invasion assay quantification and pictorial representation of the number of invaded 786-O-shControl and 786-O-shKIM-1 cells using Transwell methodology. (**B**) Invasion assay quantification and pictorial representation of the number of invaded KIM-1^pos^ and KIM-1^neg^ Renca cells using Transwell methodology. All invasion assays performed with serum free media (SFM) conditions. All membrane images were taken at × 10 magnification and scale bar = 10 µm. All data are represented as mean number of cells ± SD; n = 3 independent experiments (**A** ***p = 0.006; **B** **p = 0.0019), unpaired two-tailed *t* test).

Given the absence of KIM-1-dependent effects on RCC cell proliferation and tumour growth in vivo, we investigated the potential role of KIM-1 in altering the metastatic potential of both human and murine RCC cells. First, we used the Transwell invasion assay to compare invasion differences between shKIM-1 and shControl 786-O cells. shKIM-1 786-O cells showed significantly reduced invasive capability compared with shControl 786-O cells (Fig. 3A). Similar differences were observed with invasion differences between KIM-1^pos^ and KIM-1^neg^ Renca cells. KIM-1^pos^ Renca cells showed significantly reduced invasive capability compared with KIM-1^neg^ Renca cells (Fig. 3B). Given that the Matrigel invasion assay provides a physiologically relevant model to study the invasive potential of tumorigenic cells^25^, our data led us to conclude that KIM-1 expression in 786-O and Renca cells inhibits invasion in vitro*.* Our findings are consistent with what had been reported by Cuadros et al. who showed that 786-O cells with enhanced KIM-1 shedding (i.e. less cell-surface KIM-1) resulted in increased invasiveness in vitro^26^. However, a later study by the same group demonstrated that KIM-1 overexpression in 769-P cells (human RCC cells which do not generate tumours in vivo^27^) resulted in increased cell proliferation but inconclusive results in terms of migration- migration in KIM-1 overexpressing cells was delayed at early time points but proceeded to migrate faster than control cells at later time points^13^.

### KIM-1 inhibits the metastatic potential of RCC cells independent of adaptive immunity

**Figure 4:**
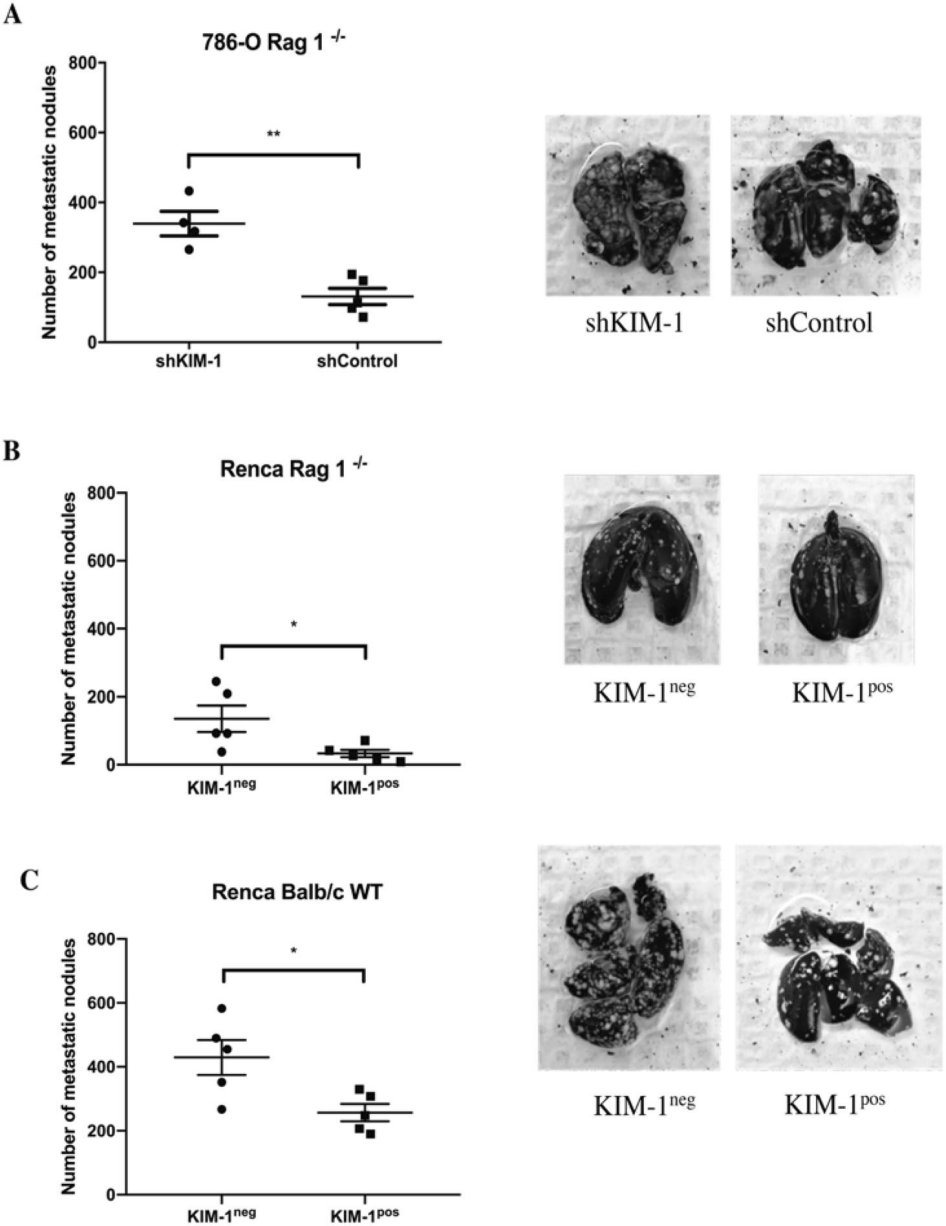
KIM-1 expression decreases metastasis of human and murine RCC cells to the lungs in immune deficient and immunecompetent mice. (**A**) Representative images and quantification of lung metastatic nodules from Rag1^−/−^ mice injected with 786-O-shControl 786-O-shKIM-1 cells (**p = 0.0013). (**B**) Representative images and quantification of lung metastatic nodules from Rag1^−/−^ mice injected with Renca KIM-1^pos^ and Renca KIM-1^neg^ cells (*p = 0.036). (**C**) Representative images and quantification of lung metastatic nodules from BALB/c (*wild type*) mice injected with 5 × 10^5^ Renca KIM-1^pos^ and Renca KIM-1^neg^ cells (*p = 0.0226). All data are represented as the mean ± SD of n = 5 mice in each group. All lungs were removed after a 17-day incubation period and stained with 15% India black ink, then subsequentially counterstained with Fekete’s solution to visualize metastatic nodules.

To test whether KIM-1 is involved in the regulation of other potential steps of the metastatic cascade, such as extravasation and colonization of target organs, we compared the formation of lung (metastatic) nodule upon intravascular injection of either 786-O or Renca cells into Rag1^−/−^ Balb/c mice^28^. Our data indicates that immune deficient Rag-1^−/−^ mice injected intravenously with shKIM-1 786-O cells exhibited significantly more metastatic lung nodules compared to mice injected with shControl 786-O cells (Fig. 4A). Moreover, we found that the immune deficient Rag-1^−/−^ mice injected intravenously with KIM-1^pos^ Renca cells developed significantly fewer metastatic lung nodules compared with mice injected with KIM-1^neg^ Renca cells (Fig. 4B). Taken together, the above data suggest that KIM-1 expression inhibits steps involved in the metastatic cascade of RCC cells including invasion and extravasation. The response to immune checkpoint inhibitors in patients with metastatic RCC underscores the importance of cells of the adaptive immune system in the disease process^29^. To determine if KIM-1 inhibited lung metastasis in the presence of the adaptive immune system in our murine cell line, we repeated injections of KIM-1^pos^ and KIM-1^neg^ Renca cells intravenously into immune-competent (*wild type*) Balb/c mice. Importantly, we once again found that KIM-1^pos^ Renca cells developed significantly fewer metastatic lung nodules compared with KIM-1^neg^ Renca cells in Balb/c mice (Fig. 4C).

To confirm our findings using an independent human RCC cell line, we silenced endogenous KIM-1 in human 769-P renal adenocarcinoma cells which also express endogenous KIM-1 using the lentiviral system used in the 786-O cells (Fig. S2A). Since 769-P cells did not form tumours in immune deficient mice (as stated above), we used our validated chorioallantoic membrane (CAM) of chicken embryo model to compare extravasation efficiency between 769-P shKIM-1 and 769-P shControl cells. In keeping with the from the intravenous model of metastasis suing the 786-O cells, we found that silencing of KIM-1 in 769-P cells significantly impeded extravasation of RCC cells from the veins of chick embryos (Fig. S2B and S2C).

Taken together, our data indicates that the KIM-1-mediated inhibition of metastasis is inherent to the cancer cells themselves, and not a by-product of the adaptive immune system targeting KIM-1 expressing cells in a more efficient way. These results could be due to loss of KIM-1 expression, which may contribute to the metastatic phenotype observed in RCC cells. However, this remains to be formally studied using human RCC metastatic tissue samples.

To explore mechanisms underlying KIM-1-dependent inhibition of metastasis, we compared the transcriptomic profiles of KIM-1^pos^ and KIM-1^neg^ Renca cells (Fig. S1). The most significant enriched gene was for the pro-metastatic GTPase, Rab27b^35^, which was downregulated 29-fold in KIM-1^pos^ vs. KIM-1^neg^ Renca cells. We were able to corroborate these data in human RCC cells—there was approximately 50% reduction in Rab27b mRNA between 786-O shKIM-1 vs. 786-O shControl cells (Fig. S6). Interestingly, high expression of Rab27b mRNA was shown to correlate with worse overall survival in patients with clear cell RCC (ccRCC) and papillary RCC (pRCC)^36^.

### KIM-1 expression in RCC patients predicts greater overall survival

**Figure 5:**
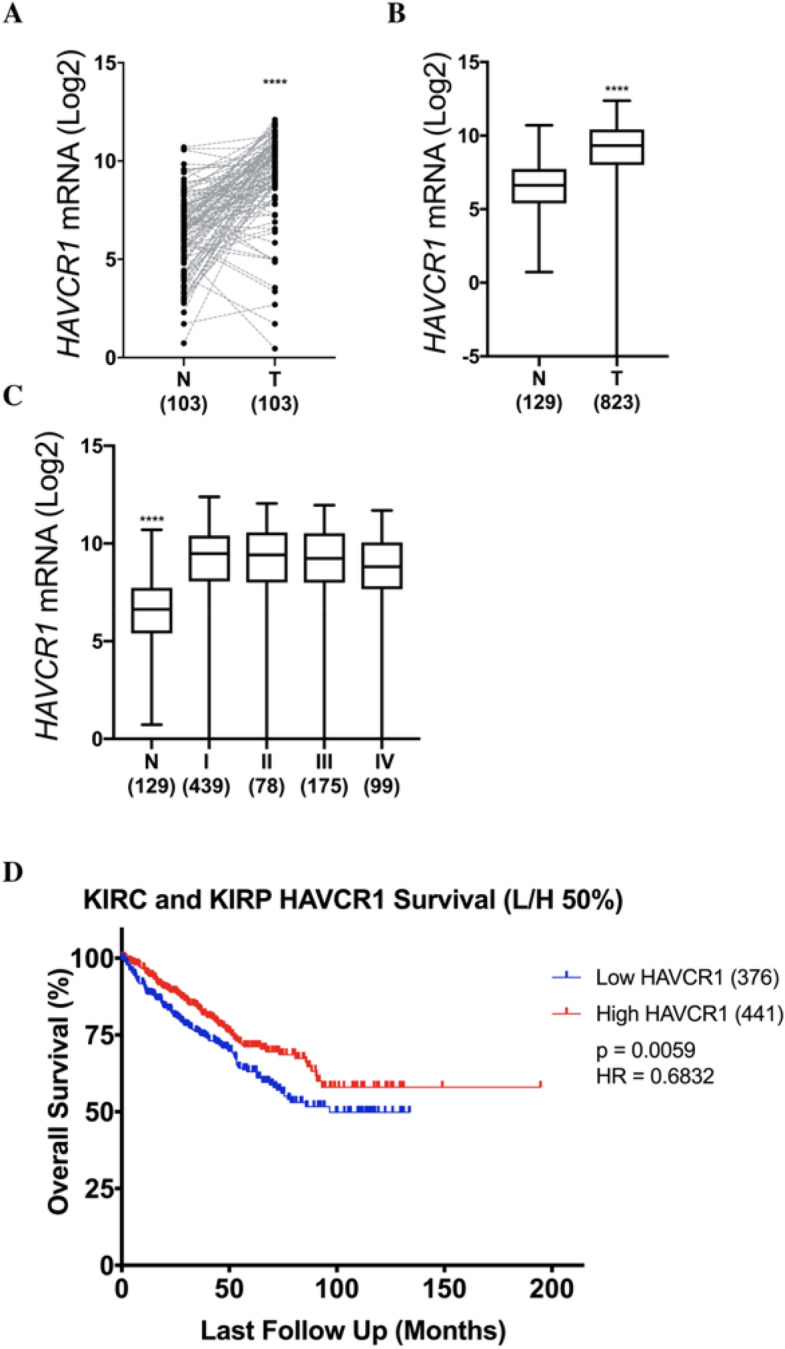
HAVCR1 (KIM-1) expression is upregulated in RCC tumours and predicts improved overall of patient with Clear Cell and Papillary RCC. (**A**) Paired comparison of adjacent tissue vs matched patient tumour tissues reveals increased *HAVCR1 *mRNA expression in tumour tissues (****p < 0.0001; Normality and Lognormality statistical analysis). (**B**) Non-paired comparison of adjacent tissues vs non-matched patient tumour tissue reveals increased *HAVCR1* (KIM-1) mRNA expression in tumour tissues (****p < 0.0001; Mann–Whitney *t* test). (**C**) RCC tumour stages vs adjacent tissues reveals increased *HAVCR1* mRNA expression in both early and late stage RCC (****p < 0.0001; Kruskal–Wallis statistical analysis). (**D**) Overall patient survival vs *HAVCR1* (KIM-1) mRNA expression using 50% low/high expression cut-offs reveals increased overall survival in patients with increased *HAVCR1* (**p = 0.0059; Kaplan–Meier statistical analysis).

Our current findings regarding KIM-1 in RCC contrast that of several groups who have claimed that KIM-1 promotes tumour growth and exacerbates cancer progression. Microvascular invasion (MVI) is defined by the invasion of cancer cells into the endothelial walls of small blood vessels and is associated with higher risks of metastases and death in patients with ccRCC^14,37^. Mijuskovic et al. (2018) showed that high degrees of MVI were associated with significantly increased expression of tumour tissue KIM-1, and that urinary KIM-1 was associated with worse disease prognoses and higher TNM staging^38^. Their study therefore suggests that KIM-1 is associated with greater risks of invasion and metastases in patients with ccRCC. We therefore sought to access the effect of KIM-1 expression on patient survival, so we analyzed The Cancer Genome Atlas (TCGA) RNAseq database for mRNA expression of *HAVCR1*. We found that RCC patients using KIRC and KIRP patient databases (both combined and individual database analysis) expressed higher *HAVCR1* mRNA within their tumour tissues compared with their normal adjacent tissues (Fig. 5A–C, Fig. S3A–C, Fig. S4A–C, Table 1), which supports our understanding that KIM-1 is highly expressed in RCC tumours^6^. When we examined the relationship between KIM-1 expression and RCC patient survival, however, we found that higher *HAVCR1* mRNA levels in patients were correlated with greater overall survival (Fig. 5D). The survival curves corresponding to the female and male patients from the same cohort are shown in figure S5. Taken together, these data suggest that although KIM-1 is highly expressed in RCC tumours, increased KIM-1 expression is predictive of greater overall patient survival.

**Figure 6:**
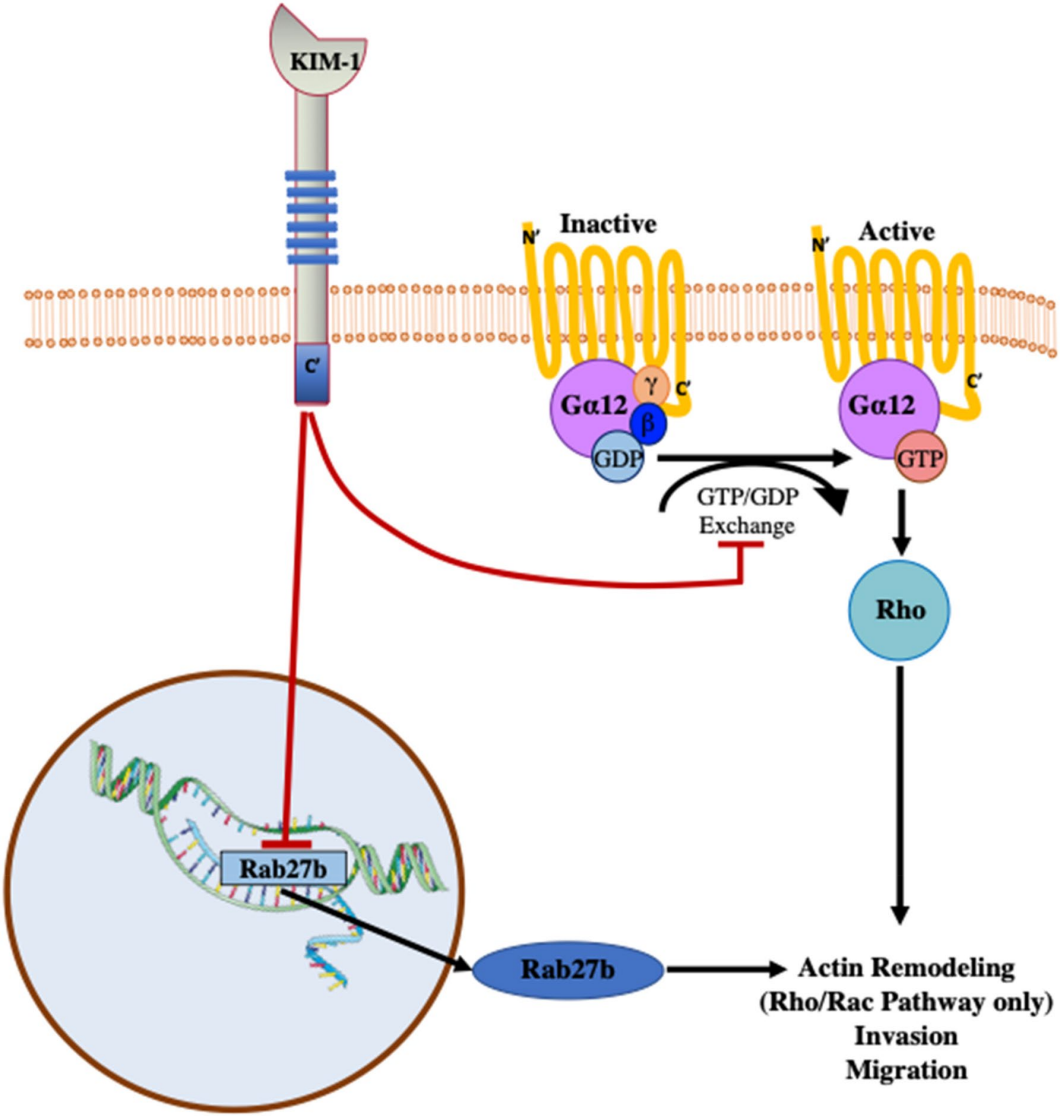
Schematic representation of the potential interaction between KIM-1 and the Rho signaling pathway in RCC cells. KIM-1 expression on RCC cells constrictively binds and inhibits Gα12 activation by blocking GTP-exchange^4^. Inactivation of Gα12 results in downstream inhibition of the small GTPase Rho which has been implicated in promoting the progression and migration of a variety of cancer cells^5,6^. Schematic representation of proposed effects how KIM-1 expression inhibits transcription of pro-metastatic Rab27b, possibly inhibiting protein effects such as invasion and metastasis of RCC cells.

Overall, our findings propose a novel role for tumour-associated KIM-1 in the pathogenesis of RCC that may be advantageous to patients. Moreover, elevated KIM-1 expression in tumour samples may serve as a positive prognostic factor for patients. Our study is limited in that the KIM-1 mRNA from the TCGA was obtained from primary tumour samples and not from metastatic tissues. Although the heterotopic tumour model used here convincingly suggested that KIM-1 does not promote tumour growth, an orthotopic (intrarenal) strategy may have offered additional insights into mechanisms of metastasis. The above conclusions may be congruent with previous findings from our group demonstrating that KIM-1 binds to and suppresses its activity of Gα_12_^10,18^. Gα_12_ has been demonstrated to promote metastasis in RCC via its activation by lysophosphatidic acid G-protein-coupled receptor^30,31^. Gα_12_ has also been shown to promote the expression of TGF-β1 through a Rho/Rac-dependent pathway, which ultimately leads to the induction of invasion (Fig. 6)^31^. Rho-GTPases are downstream effectors of Gα_12_ and are crucial to the processes of actin remodeling and invasion^31–34^.

## Supplementary Information

Supplementary Information.
