# Supplementary material for: Kidney injury molecule-1 inhibits metastasis of renal cell carcinoma
Source: Sci Rep. 2021 Jun 4;11:11840. doi: 10.1038/s41598-021-90919-8 (PMC8178330; doi:10.1038/s41598-021-90919-8)
Supplement: Supplementary file 1 — Supplementary Information. [file 41598_2021_90919_MOESM1_ESM.docx]

**Kidney injury molecule-1 Inhibits metastasis of renal cell carcinoma**

Jasper C. Lee^1,2, #^, Demitra M. Yotis^1,#^ , Ji Yun Lee,^1,2^ Marie A. Sarabusky^1,2^, Bradly Shrum^2^, Audrey Champagne^3^, Ola Z. Ismail^1^, Elena Tutunea-Fatan^2^, Hon S. Leong^4^, Lakshman Gunaratnam^1,2,5*^

^1^Department of Microbiology and Immunology, Schulich School of Medicine and Dentistry, Western University, London, Ontario, Canada

^2^Matthew Mailing Centre for Translational Transplant Studies, Lawson Health Research Institute, London, Ontario, Canada

^3^Centre de recherche du CHU de Québec-Université Laval , CHU de Québec-Université Laval, Quebec City, Quebec, Canada

^4^Sunnybrook Health Sciences Centre, Toronto, Ontario, Canada

^5^Division of Nephrology, Department of Medicine, Schulich School of Medicine and Dentistry, Western University, London, Ontario, Canada

# These authors contributed equally

**Supplementary information**

**Supplementary methods**

**769-P cell lines and shRNA knockdown of endogenous KIM-1**

Human 769-P renal cell adenocarcinoma (CRL-1933) cells were purchased from American Type Tissue Collection (ATCC) and cultured as previously described^1,2^. Lentiviral particles containing three human KIM-1-specific constructs encoding shRNA (sc-61691; Santa Cruz) were used to knockdown KIM-1 in the 769-P cell line (herein referred to as 769-P shKIM-1). Control transduction was done using scrambled shRNA lentiviral particles (769-P shControl) (sc-108080; Santa Cruz). Polyclonal clones were selected post transfection, and stable cell lines expressing the respective shRNA were isolated via puromycin.

**Chick embryo model and extravasation assay**

The quantification of cancer cell extravasation using the experimental chorioallantois membrane (CAM) model is described in detail elsewhere ^3^. Briefly, 769-P shKIM-1 and 769-P shControl cells were fluorescently labeled with CellTracker Green^TM^ dye, and 0.5 x 10^5^ cells were injected into the veins of chick embryos at day 13 of gestation. Extravasated cells were enumerated at filter windows at 0 h and 24h via confocal microscopy. Embryos were injected with lectin-DyLight 649 (purple) and Dextran-Alexa 555 (red) to reveal endothelial walls and vessel lumen to visualize intravascular or extravascular positioning of 769-P cells (green). Intravital images were captured using confocal resonance scanning microscopy. The injected 769-P cells extravasate into the underlying stroma, which is not labeled by the lectin, providing a clear distinction between intravascular and extravasated cancer cells. Extravasation efficiency was expressed as a percentage of # of cells at time zero divided by the #ofextravasated cells at 24 h^3^.

**Supplementary Tables and Figure Legends**


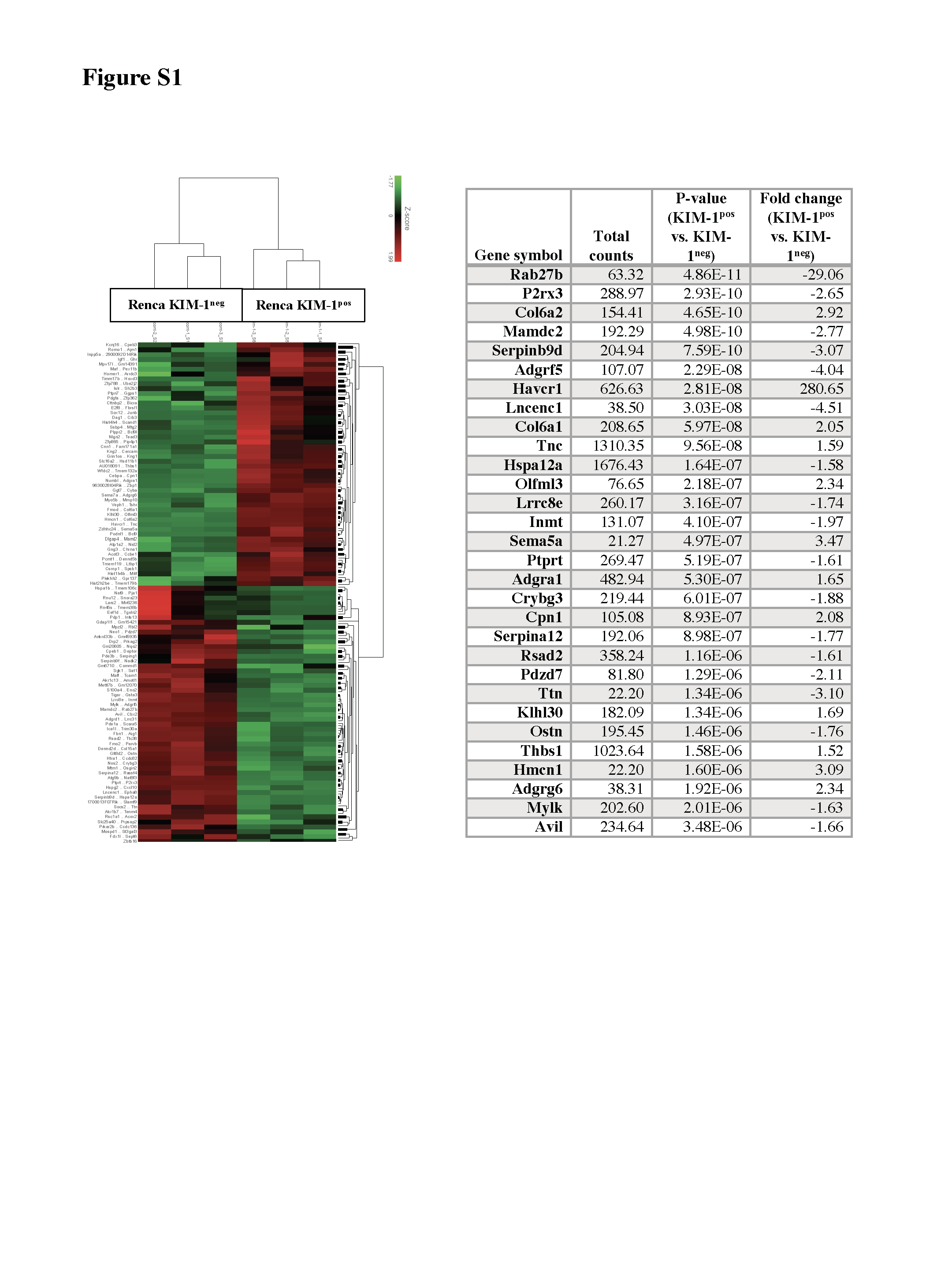


**Figure S1. Illumina RNA-seq transcriptomic analysis of Renca KIM-1^pos^ and Renca KIM-1^neg^ cell lines.**

**A,** Heat map generated from RNA sequences of both Renca KIM-1^pos^ vs Renca KIM-1^neg^ cell lines, displaying genes with significant differences in expression, (p>/=0.05). Genes with decreased expression marked as green, and increased expression marked as red. **B**, Top 30 significantly enriched genes listed from Renca KIM-1^pos^ vs. Renca KIM-1^neg^ cell lines. Gene list extrapolated from enriched gene analyses displayed in heat map.

**Figure S2. KIM-1 expression decreases extravasation capability of human 769-P and murine Renca cell lines.**

**A,** Western blot analyses confirming successful shRNA knockdown of exogenous KIM-1 in 769-P shKIM-1, but not 769-P shControl cell lines. Experimental chorioallantois membrane (CAM) model where, 0.5 x 10^5^ fluorescently labeled 769-P shKIM-1 and shControl cells or KIM-1^pos^ and KIM_1^neg^ Renca cells were injected into the vein of chick embryo at day 13 of gestation. Fluorescently labeled, extravasated cells were enumerated at 0 h and 24h via confocal microscopy for **B,** 769-P and **C,** Renca cells (769-P: ***, p < 0.0001 and Renca: * = p < 0.05). The mean of extravasation efficiency in percentage per group ± SEM.

**Figure S3. Increased expression of HAVCR1 mRNA in Clear Cell Renal Carcinoma (KIRC) tumours compared to adjacent normal tissue.**

**A,** Paired comparison of normal adjacent tissue vs matched patient tumour tissues reveals increased *HAVRC1* mRNA expression in tumour tissues, (****, P<0.0001; Normality Lognormality statistical analysis). **B,** Non-paired comparison of normal adjacent tissues vs non-matched patient tumour tissue reveals increased *HAVCR1* mRNA expression in tumour tissues, (****, P<0.0001, Mann-Whitney *t*-test). **C**, Ratio of tumour and normal adjacent tissue vs *HAVRC1*(KIM-1) mRNA expression (one sample *t* and Wilcoxon test). **D,** RCC tumour stages vs matched normal adjacent tissues reveals increased *HAVCR1* mRNA expression in both early and late stage RCC (****, P<0.0001, Kruskal-Wallis statistical analysis).

**Figure S4. Increased expression of HAVCR1 mRNA in Papillary Renal Carcinoma (KIRP) tumours compared to adjacent normal tissue.**

**A,** Paired comparison of normal adjacent tissue vs matched patient tumour tissue reveals increased *HAVRC1* mRNA expression in tumour tissues, (**, P=0.0032, Normality and Lognormality statistical analysis). **B,** Non-paired comparison of normal adjacent tissues vs non-matched tumour tissue reveals increased *HAVCR1* mRNA expression in tumour tissues, (****, P<0.0001, Mann-Whitney *t*-test). **C**, Ratio of tumour and normal adjacent tissue vs *HAVRC1* mRNA expression (one sample *t* and Wilcoxon test). **D,** RCC tumour stages vs matched normal adjacent tissues reveals increased *HAVCR1* mRNA expression in both early and late stage RCC (****, P<0.0001, Kruskal-Wallis statistical analysis).

**Figure S5. Survival benefit of HAVCR1 mRNA expression in female and male patients with Clear Cell Renal Carcinoma (KIRC) and Papillary Renal Carcinoma (KIRP).**

**A,** Female patient survival vs *HAVCR1* mRNA expression using 50% low/high expression cut-offs reveals a trending increased overall survival in female patients with increased *HAVCR1*, (NS, p = 0.0507; Kaplan-Meier statistical analysis). **B,** Male patient survival vs *HAVCR1* mRNA expression using 50% low/high expression cut-offs reveals increased overall survival in male patients with increased *HAVCR1*, (*, p = 0.0446; Kaplan-Meier statistical analysis).

**Figure S6. Rab27b mRNA expression in 786-O shKIM-1 and 786-O shControl RCC cells.**

**A,** RT-qPCR of 786-O cell lines normalized to housekeeping gene (β-actin), showing decreased mRNA expression of pro-metastatic Rab27b in 786-O shKIM-1, but not 786-O shControl cell lines (***, p = 0.0002). **B,** Schematic representation of how tumour KIM-1 may potentially suppress Rab27b. KIM-1 expression may downstream inhibit the transcription of Rab27b, resulting in Rab27b pro-metastatic protein effects – increased invasion and metastasis – to be inhibited.

**Figure S7. Uncropped western blots of 786-O shKIM-1 and shControl, 769-P shKIM-1 and shControl, and Renca KIM-1^neg^ and KIM-1^neg^ cell lines displaying KIM-1 expression.**

**A,** Developed film of various cell lines, including human 786-O shKIM-1 and 786-O shControl cells staining for human KIM-1 at ~90kDa. Subsequential developed film staining for housekeeping gene, human β-actin at ~40kDa. **B,** Developed film of various cell lines, including human 769-P shKIM-1 and 769-O shControl cells staining for human KIM-1 at ~90kDa. Subsequential developed film staining for housekeeping gene, human β-actin at ~40kDa. **C,** Membrane image of murine Renca KIM-1^neg^ and KIM-1^pos^ cells staining for murine KIM-1 at ~70-80kDa along with housekeeping gene, murine GAPDH at ~37kDa.

**Supplementary information - References**

1 Gandhi, R. *et al.* Accelerated receptor shedding inhibits kidney injury molecule-1 (KIM-1)-mediated efferocytosis. *Am J Physiol Renal Physiol* **307**, F205-221, doi:10.1152/ajprenal.00638.2013 (2014).

2 Ichimura, T. *et al.* Kidney injury molecule-1 is a phosphatidylserine receptor that confers a phagocytic phenotype on epithelial cells. *J Clin Invest* **118**, 1657-1668, doi:10.1172/JCI34487 (2008).

3 Kim, Y. *et al.* Quantification of cancer cell extravasation in vivo. *Nat Protoc* **11**, 937-948, doi:10.1038/nprot.2016.050 (2016).

4 Ismail, O. Z. *et al.* Kidney injury molecule-1 protects against Galpha12 activation and tissue damage in renal ischemia-reperfusion injury. *Am J Pathol* **185**, 1207-1215, doi:10.1016/j.ajpath.2015.02.003 (2015).

5 Sahai, E. & Marshall, C. J. RHO-GTPases and cancer. *Nat Rev Cancer* **2**, 133-142, doi:10.1038/nrc725 (2002).

6 Narumiya, S., Tanji, M. & Ishizaki, T. Rho signaling, ROCK and mDia1, in transformation, metastasis and invasion. *Cancer Metastasis Rev* **28**, 65-76, doi:10.1007/s10555-008-9170-7 (2009).
